# CPT1A mediates the succinylation of SP5 which activates transcription of PDPK1 to promote the viability and glycolysis of prostate cancer cells

**DOI:** 10.1080/15384047.2024.2329372

**Published:** 2024-03-17

**Authors:** Shufeng Liu, Xiaoguang Chen, Liqi Zhang, Bo Lu

**Affiliations:** a Medical Department, Xiangyang Integrated Traditional and Western Medicine Hospital, Xiangyang, Hubei, China; b Urology Department, Xiangyang Integrated Traditional and Western Medicine Hospital, Xiangyang, Hubei, China; c Laboratory Department, Xiangyang Integrated Traditional and Western Medicine Hospital, Xiangyang, Hubei, China

**Keywords:** Prostate cancer, CPT1A, SP5, PDPK1, succinylation, transcription

## Abstract

Succinylation modification involves in the progression of human cancers. The present study aimed to investigate the role of CPT1A, which is a succinyltransferase in the progression of prostate cancer (PCa). CCK-8 was used to detect the cell viability. Seahorse was performed to evaluate the cell glycolysis. Luciferase assay was used to detect the transcriptional regulation. ChIP was performed to assess the binding between transcriptional factors with the promoters. Co-IP was used to assess the binding between proteins. We found that CPT1A was highly expressed in PCa tissues and cell lines. Silencing of CPT1A inhibited the viability and glycolysis of PCa cells. Mechanistically, CPT1A promoted the succinylation of SP5, which strengthened the binding between SP5 and the promoter of PDPK1. SP5 activated PDPK1 transcription and PDPK1 activated the AKT/mTOR signal pathway. These findings might provide novel targets for the diagnosis or therapy of prostate cancer.

## Introduction

Prostate cancer (PCa) is the third leading cause of cancer related death and is the fifth most common cancer worldwide.^1,2^ Although the incidence of PCa is declining, there are still one million new cases worldwide each year.^3^ The 5-year survival rate of early PCa is more than 60%.^4^ Surgery and radiation are the curative treatments for localized disease; however, adverse effects seriously affect the quality of life.^5^ Growing evidence has highlighted the important role of androgen receptors (AR) in the onset and progression of PCa, so androgen deprivation therapy alone is the standard treatment for metastatic PCa.^6^ Unfortunately, the 5-year survival rate for advanced PCa decreases to 30%.^7,8^ This suggests that we need to have a deeper understanding of the pathogenesis of PCa and explore new therapeutic strategies.

PCa has been found to exhibit high rates of de novo fatty acid synthesis driven by activation of the AR.^9^ Fatty acid synthesis is a synthetic process whereby two carbons are added in a stepwise manner to a growing chain that can be further modified by addition of substituents on the hydrocarbon chain such as the succinyl group. Increased studies have shown that succinylation modification promotes or inhibits the progression of various cancers by regulating different substrate targets or signaling pathways, such as liver cancer, breast cancer, gastric cancer and PCa.^10–13^ However, the modification pathway and regulation mechanisms of succinylation modification in the occurrence and development of PCa remain unknown. Succinylation modification refers to the covalent binding of succinyl groups by succinyl-coenzyme A (CoA) donors to lysine residues, which is a newly discovered post-translational modification of proteins.^14,15^ It exists widely in cells and participates in a variety of biological processes.^16^ Succinylation is involved in the tricarboxylic acid cycle, amino acid metabolism and fatty acid metabolism by regulating protease activity and gene expression.^17^ Therefore, it is of great significance to explore the role of succinylation in PCa.

Carnitine palmitoyl transferase 1 (CPT1) controls the rate-limiting step of fatty acid oxidation.^18^ It promotes the passage of fatty acids into the mitochondria by loading fatty acyl groups onto carnitine. Fatty acid metabolism supports the uncontrolled growth of cancer cells.^19^ The rate of fatty acid synthesis is high under AR activation conditions, which plays an important role in facilitating PCa invasion.^9^ The CPT1 family consists of three subtypes including CPT1A, CPT1B and CPT1C.^20^ Recently, lysine acetyltransferase 2A (KAT2A) and carnitine palmitoyltransferase 1A (CPT1A) have been determined to have lysine succinyltransferase activities.^21,22^ A previous study reports that CPT1A knockdown suppresses PCa cell viability, colony formation, and sphere formation, and impedes tumor growth.^23^ Moreover, CPT1A promotes PCa cell growth in an androgen-dependent manner.^24^ Androgens regulate CPT1A activity, and inhibition of CPT1A reduces PCa cell growth and AR expression.^25^ However, whether CPT1A regulates glycolysis of PCa cells remains unknown.

Here, we aimed to explore the role of CPT1A in PCa development. Mechanistically, we tried to find out the substrate proteins modulated by CPT1A to elucidate the mechanism of CPT1A in PCa progression. We hope these findings might provide novel strategies for PCa therapy.

## Materials and methods

### Tissue specimens

Paired clinical primary PCa tissues and paracarcinoma non-tumor tissues (*n* = 35) were acquired from patients with PCa who were diagnosed at the Xiangyang Integrated Traditional and Western Medicine Hospital. All fresh tissues were stored at −80°C before use. This study was approved by the Xiangyang Integrated Traditional and Western Medicine Hospital. Written informed consent was obtained from all the patients.

### Cell culture

HEK-293T cells, normal human epithelial prostate PNT2 cells and the PCa cell lines including DU145, LNCap, 22Rv1 and VCaP were purchased from ATCC (USA) and were maintained in Dulbecco’s modified eagle medium containing 10% fetal bovine serum and 1% penicillin/streptomycin (all from Thermo Fisher Scientific, USA). The cells were cultured at 37°C with 5% CO_2_ and 95% air. The culture medium was replaced every 2 days.

### Cell transfection

The cells were seeded in 6-well plates and cultured until they reached 70% confluence. siRNAs such as si-CPT1A 1#, si-CPT1A 2#, si-SP5, si-nc were purchased from General Biocompany (Anhui, China). The CPT1A overexpressing vector (oe-CPT1A), PDPK1 overexpressing vector (oe-PDPK1) and the empty vector (oe-nc) were purchased from GenePharma (Shanghai, China). LNCap, DU145, 22Rv1, and HEK-293T cells were seeded in 6-well plates and transiently transfected with 100 nM of those plasmids using Lipofectamine 3000 (Invitrogen, CA, USA) following the protocols. The culture medium was changed with new complete medium 6 h after transfection. Forty-eight hours later, the transfected cells were harvested.

### RNA isolation and real-time PCR (qPCR)

TRIzol reagent (Invitrogen, USA) was used to isolate total RNA. After detecting RNA purity at A260/280, 1 μg of total RNA was reverse transcribed to cDNA using the SuperScript IV First Strand cDNA synthesis kit (Thermo Fisher Scientific, USA). qPCR was performed using the AceQ qPCR SYBR Green Master Mix (Vazyme, China) on a real-time PCR system (CFX96; Bio-Rad, USA). The relative expression of mRNA (fold) was calculated using the 2^−ΔΔCt^ method by normalizing to GAPDH.

### Dual-luciferase assay

The wild-type (WT) and mutant (MUT) promoters of PDPK1 were cloned into a pGL3 vector (U47295, Promega, Madison, WI, USA). Cotransfection of wt-PDPK1 or mut-PDPK1 and si-nc or si-SP5 in HEK-293T cells was performed using Lipofectamine 3000. Firefly/Renilla luciferase activities were examined using a Dual-Lucy Assay Kit (D0010, Solarbio, Beijing, China) after 24 h on a SpectraMax M5 microplate reader (Molecular Devices, Shanghai, China).

### Cell counting kit-8 (CCK-8) analysis

The DU154 and LNCap cells were seeded onto 96-well plates and incubated for 48 h. Subsequently, the CCK-8 solution (10 μL) was added into the culture medium for 4 h, and the OD value at 450 nm was detected using a microplate reader (Elx808, BioTek, Biotek Winooski, Vermont, USA).

### Measurement of glucose uptake and lactate production

A Screen Quest Fluorimetric Glucose Uptake Assay Kit (AAT36500, AAT Bioquest) was used to detect the glucose uptake of the DU154 and LNCap cells according to the instructions. The detection of lactate in DU154 and LNCap cells was performed according to the instructions of the LA detection kit (BC2230, Solarbio, Beijing, China).

### Extracellular acidification rate (ECAR) and oxygen consumption rate (OCR) analysis

DU154 and LNCap cells were seeded into XF96 culture plates for ECAR and OCR analysis using the XF96 extracellular flux analyzer (Seahorse Bioscience). Glucose, oligomycin A, and 2-DG were automatically added for ECAR analysis. Oligomycin A, carbonyl cyanide 4-(trifluoromethoxy) phenylhydrazone (FCCP), and antimycin A and rotenone (Rote/AA) were automatically added for OCR analysis.

### Co-immunoprecipitation (Co-IP)

Co-IP was used to detect the interaction between CPT1A and SP5 in HEK-293T cells. The cells were lysed on ice using RIPA (Beyotime Biotechnology, Shanghai, China) buffer containing protease inhibitors for 30 min. The supernatant was collected and a small amount of it was taken as the input group. CPT1A or lgG antibody (Thermo Fisher, 2 μg) was added into the remaining supernatant for incubation overnight at 4°C. Afterward, 10 µL of protein A agarose beads (Thermo Fisher) was pre-treated by washing three times with appropriate lysis buffer (Beyotime) and then added to the cell lysate and antibody complex, and slowly shaken at 4°C for 2 h to make the antibody conjugated with the protein A agarose beads. After the immunoprecipitation reaction, the complex was centrifuged at 3,000 rpm for 3 min at 4°C. Next, the supernatant was discarded and the agarose beads were washed with 1 mL of lysis buffer three times. Finally, 15 μl of 2 × SDS loading buffer (Beyotime) was added and boiled for 5 min. The precipitated protein was then analyzed using western blot assay as described above.

### Chromatin immunoprecipitation (ChIP) assay

The HEK-293T cells were cleaved after being fixed with formaldehyde. A chromatin extraction kit (AB117152, Abcam) was used to prepare chromatin. Diagenode Bioruptor Pico Ultrasonic crusher (Belgium) was used to shear chromatin to the average size of 250–300 bp (about 25 cycles). The ChIP Kit Magnetic-One Step (ab156907, Abcam) was used for ChIP analysis. Chromatin was mixed with antibodies or IgG in the CHIP buffer according to the manufacturer’s instructions. The mixture was washed and then eluted with DNA release buffer and protease K. The samples were cross-linked in reverse (15 min at 65°C, then 10 min at 95°C). After DNA was purified, the enrichment of the target gene was analyzed by qPCR.

### Statistical analysis

All data were acquired from three independent replicate experiments and represented as mean ± SD. One-way ANOVA followed by Tukey’s post hoc test was used to analyze differences among multiple groups, and Student’s t-test was performed to assess differences between the two groups. *p* < .05 means a statistically significant difference. Statistics were analyzed using the GraphPad Prism software (version 8.0; GraphPad Software, USA).

## Results

### CPT1A expression is upregulated in PCa

**Figure 1.**
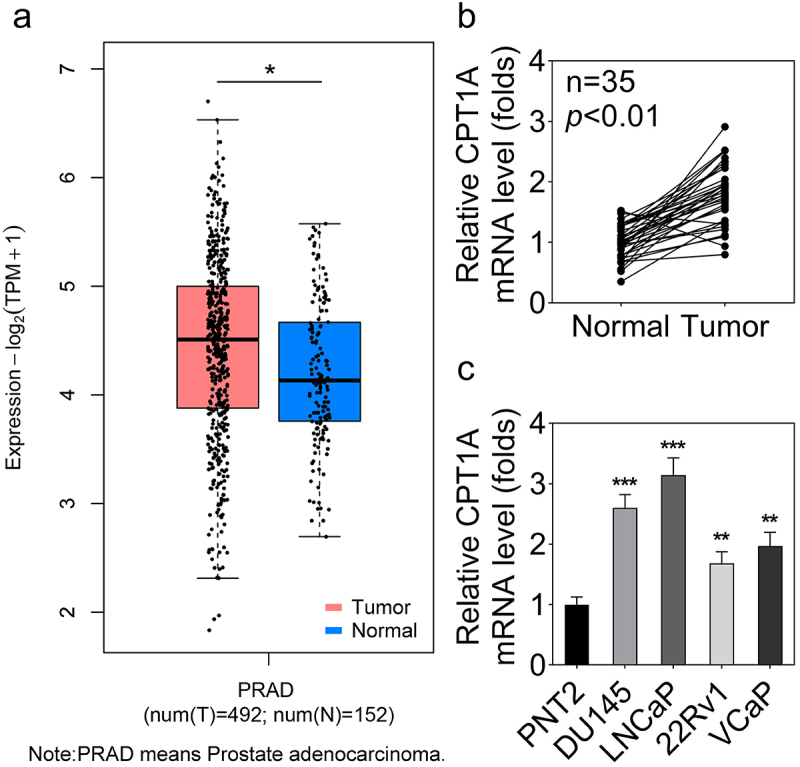
CPT1A is highly expressed in PCa tissues and cell lines. (a) GEPIA was used to analyze the dysregulated expression of CPT1A in PCa. PRAD: prostate adenocarcinoma. T: tumor and N: normal. (b) The expression of CPT1A in PCa tissues and the normal tissues was detected by qPCR. (c) The expression of CPT1A in PCa cell lines and the prostate epithelial cell line (PNT2) was detected by qPCR. *p<.05 in (A). **p < .01, ***p < .001 vs PNT2 group.

We first evaluated CPT1A levels in PCa tissues and cells. The bioinformatics analysis based on the Gene Expression Profiling Interactive Analysis (GEPIA) database indicated up-regulation of CPT1A mRNA in PCa (Figure 1(a)). qPCR data showed that CPT1A expression was significantly higher in PCa tissues than that in para-cancerous tissues (Figure 1(b)). Furthermore, CPT1A expression was significantly higher in PCa cells than in normal PNT2 cells (Figure 1(c)).

### Interference with CPT1A suppresses the viability and glycolysis of PCa cells

**Figure 2.**
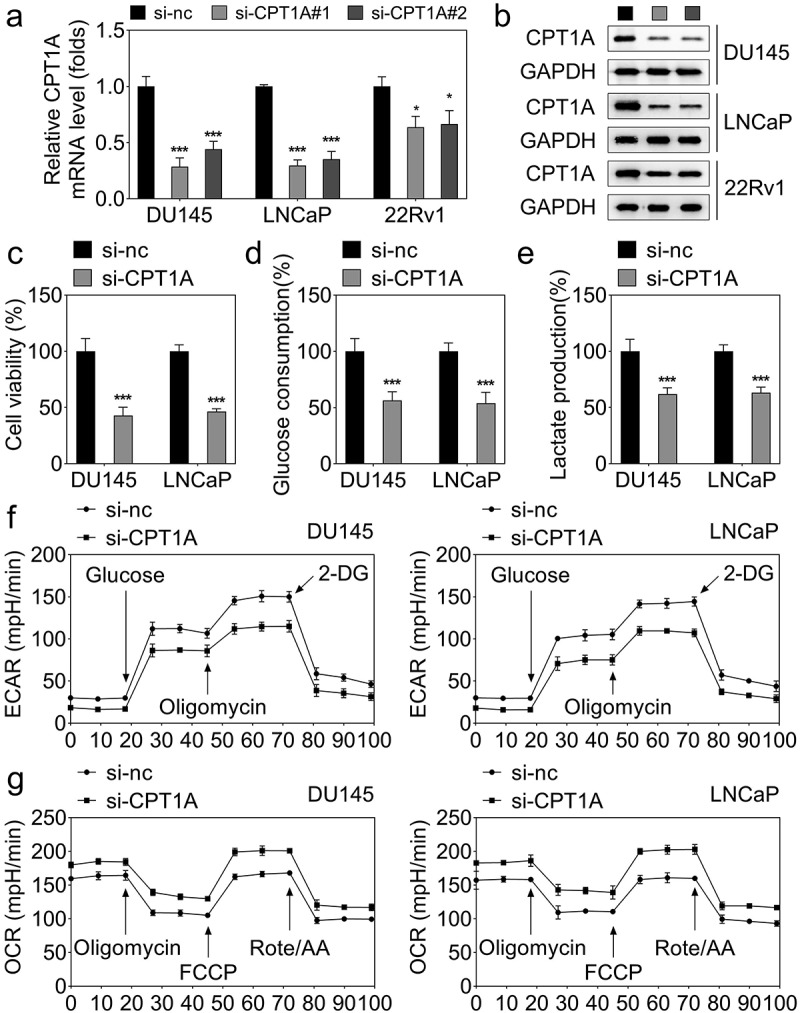
Silencing of CPT1A inhibits the viability and glycolysis of PCa cells. (a) mRNA expression of CPT1A after transfection. (b) Protein levels of CPT1A after transfection. (c) Cell viability was evaluated by CCK-8 assay. (d,e) glucose consumption and lactate production were measured using commercial kits. (f,g) Seahorse was used to detect the glycolysis of PCa cells after transfection. *p < .001 vs si-nc group.

CPT1A expression was markedly downregulated in the si-CPT1A#1 and si-CPT1A#2 transfected PCa cells (Figure 2(a,b)). DU145 and LNCaP cells with lower CPT1A expression were selected for the determination of the biological behaviors. Due to the lower CPT1A expression after si-CPT1A#1 transfection than si-CPT1A#2 transfection, we chose si-CPT1A#1 transfected cells for subsequent experiments. We found that knockdown of CPT1A dramatically inhibited cell viability (Figure 2(c)), glucose consumption (Figure 2(d)), and lactate production (Figure 2(e)) of PCa cells. Moreover, the seahorse analysis indicated that CPT1A silencing reduced the glycolysis capability of PCa cells (Figure 2(f,g)).

### CPT1A promotes the expression of PDPK1 without succinylation modulation

**Figure 3.**
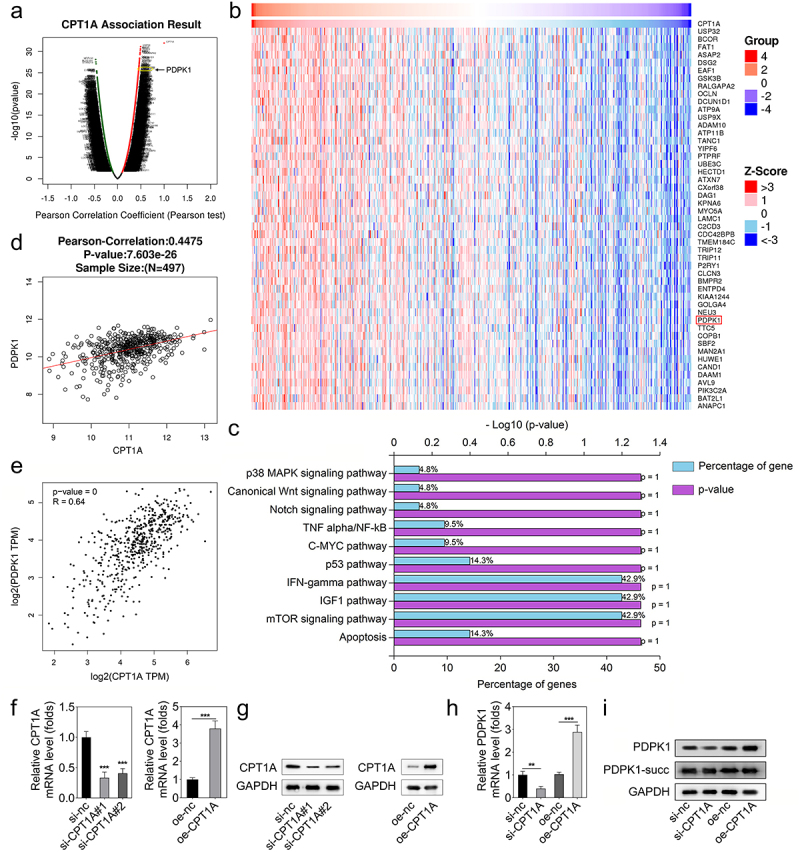
CPT1A promotes the expression of PDPK1 without succinylation modulation. (a,b) the LinkedOmics database was used to screen the positively correlated genes of CPT1A. (c) Enrichment analysis was performed. (d,e) the LinkedOmics and GEPIA databases were used to further confirm the relation between CPT1A and PDPK1. (f) qPCR was performed to detect CPT1A RNA expressions after transfection. (g) Western blot was conducted to measure CPT1A protein levels after transfection. (h) qPCR was used to measure the expression of PDPK1 mediated by CPT1A. (I) Western blot was used to detect the succinylation and expression of PDPX1. *p < .01, **p < .001.

The data acquired from the LinkedOmics database indicated the genes whose expression is positively related to CPT1A expression (Figure 3(a,b)). PDPK1 was one of the positively related genes to CPT1A. Moreover, KEGG enrichment analysis showed that those genes could be enriched to IFN-gamma, IGF-1, and mTOR signaling pathways (Figure 3(c)). PDPK1 was included in these three pathways. Thus, we chose PDPK1 for further investigation. The analysis based on the data of the LinkedOmics and GEPIA databases confirmed that PDPK1 expression was positively related to CPT1A (Figure 3(d,e)). Then, we transfected si-CPT1As and CPT1A overexpressing vector into HEK293T cells, and found that CPT1A expression was reduced after si-CPT1A#1 and si-CPT1A#2 transfection, and CPT1A expression was promoted by CPT1A overexpression (Figure 3(f,g). After transfection, we found that CPT1A silencing inhibited PDPK1 expression while CPT1A overexpression promoted PDPK1 expression (Figure 3(h)). Western blot also showed that CPT1A silencing inhibited protein expression of PDPK1 while CPT1A overexpression suppressed that. However, the succinylation of PDPK1 was not influenced (Figure 3(i)).

### CPT1A binds with SP5 to modulate the transcription of PDPK1

**Figure 4.**
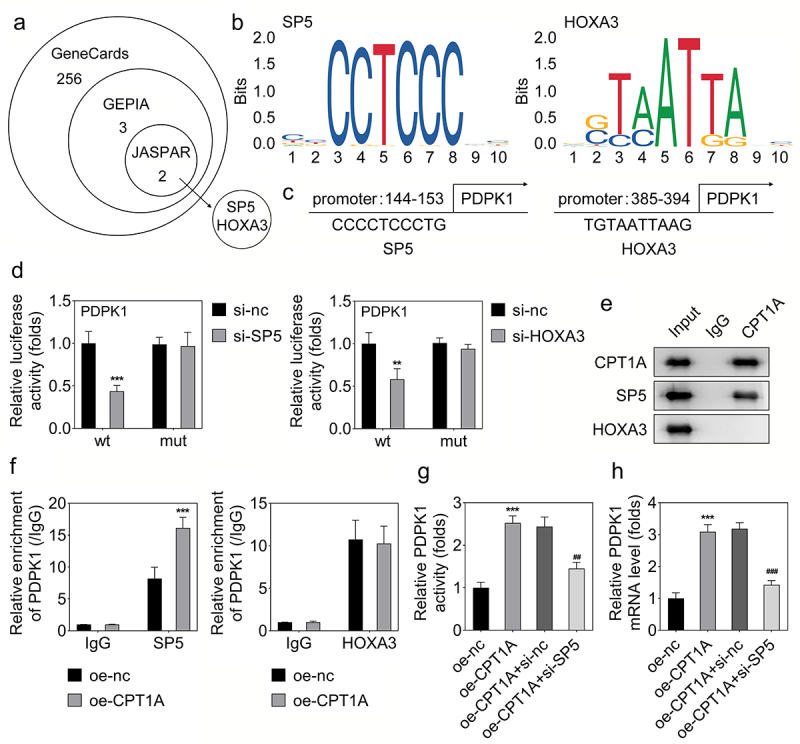
CPT1A binds with SP5 to modulate the transcription of PDPK1. (a) Genecards, GEPIA and JASPAR were used to screen the transcriptional factors that regulated the transcription of PDPK1. (b,c) the sites in the promoter of PDPK1 potentially bind with SP5 or HOXA3 was shown. (d) Luciferase assay was carried out to check whether SP5 or HOXA3 could regulate the transcription of PDPK1. (e) CO-IP was performed to detect the binding between CPT1A with SP5 or HOXA3. (f) ChIP assay was used to investigate the binding between SP5 or HOXA3 with the promoter of PDPK1 after CPT1A overexpression. (g) Luciferase assay was performed to assess the luciferase activity of PDPK1 in each group. (H) qPCR was used to detect the RNA expression. *p < .001 vs oe-nc, ## p < .01, ### p < .001 vs oe-CPT1A+si-nc.

As we found that CPT1A regulates the RNA expression of PDPK1 but doesn’t influence its succinylation, we speculated that CPT1A might interact with some transcriptional factors to modulate the transcription of PDPK1. Thus, we predicted the potential transcriptional factors of PDPK1 using Genecards, GEPIA and JASPAR software. SP5 and HOXA3 were predicted as the potential transcriptional factors of PDPK1 (Figure 4(a)). The potential binding sites in the PDPK1 promoter with SP5 and HOXA3 were shown (Figure 4(b,c)). Luciferase assay revealed that both SP5 and HOXA3 can regulate the transcription of PDPK1 (Figure 4(d)). However, co-IP assay indicated that CPT1A only directly bound with SP5 but not HOXA3 (Figure 4(e)). ChIP assay showed that overexpression of CPT1A promoted the binding of SP5 with the promoter of PDPK1, but didn’t influence the binding of HOXA3 with the PDPK1 promoter (Figure 4(f)). Rescue experiments revealed that SP5 silencing inhibited the effect of CPT1A on promoting the activity and expression of PDPK1 (Figure 4(g,h)). These results confirmed that CPT1A binds with SP5 and promotes the transcription of PDPK1.

### CPT1A regulates the succinylation of SP5 at K391 and promotes its transcriptional function

**Figure 5.**
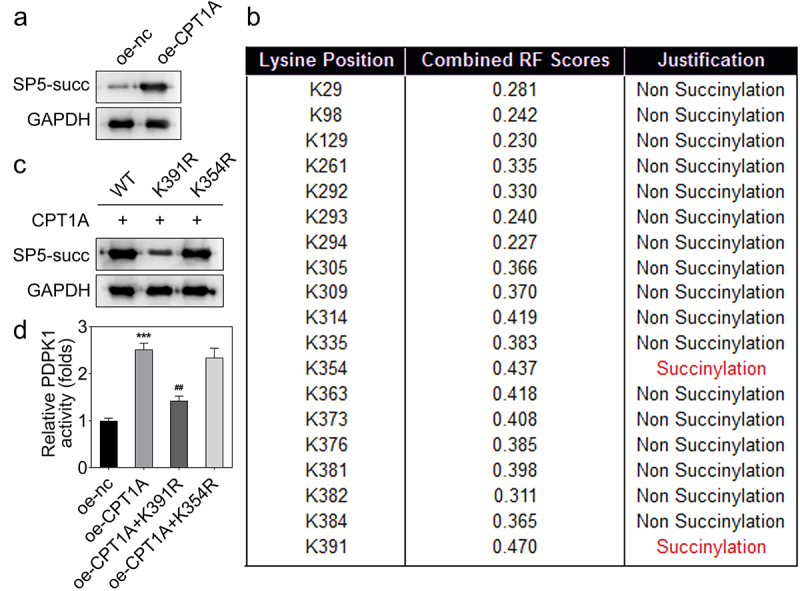
CPT1A mediates the succinylation of SP5. (a) The succinylation level of SP5 after CPT1A overexpression was detected by western blot. (b) The potential succinylation sites in SP5 were predicted using the SuccinSite database. (c) Western blot analysis was used to evaluate the succinylation level of SP5 after transfection of WT, K391R or K354R expressing vectors. (d) The activity of PDPK1 was evaluated in each group. *p < .001 vs oe-nc, ## p < .01 vs oe-CPT1A.

To elucidate the precise mechanism, we assessed whether SP5 could be succinylated by CPT1A. We found that CPT1A overexpression promoted the succinylation of SP5 (Figure 5(a)). Then, we tried to find out the succinylation sites in SP5 that were modulated by CPT1A. Using bioinformatics analysis, we found two potential succinylation sites in SP5 sequence including K391 and K354 (Figure 5(b)). To verify whether these two sites were succinylated, we constructed the mutant plasmids including K391R and K354R. The western blot assay indicated that K391R inhibited the expression and succinylation of SP5 (Figure 5(c)). In addition, we found that co-transfection with oe-CPT1A and K391R reduced the activity of PDPK1 while K354R had no this effect (Figure 5(d)). These results indicated that CPT1A binds with the K391 of SP5 and promotes the activity of PDPK1.

### PDPK1 regulates the AKT/mTOR signal pathway

**Figure 6.**
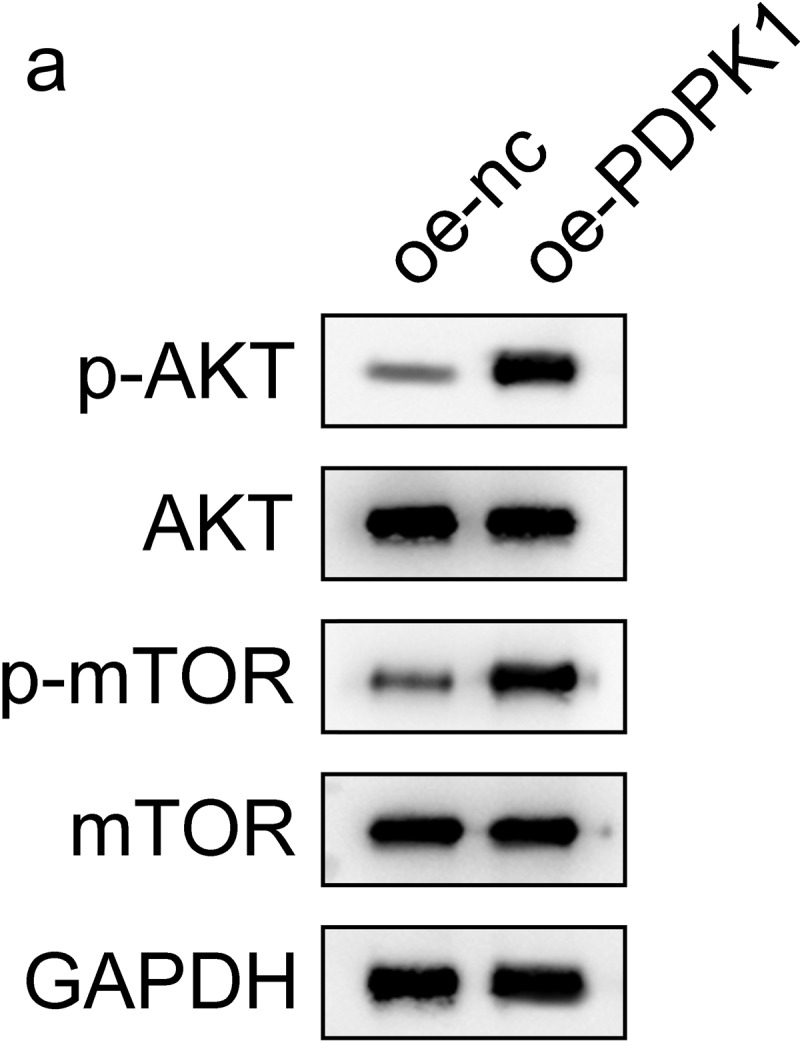
PDPK1 regulates the AKT/mTOR signal pathway. (a) Western blot was used to detect the expression and phosphorylation of AKT and mTOR.

To identify how PDPK1 mediates the effect of CPT1A on the viability and glycolysis of PCa cells, we evaluated the expression and phosphorylation of AKT and mTOR. The results revealed that PDPK1 activated the AKT/mTOR signaling (Figure 6(a)).

### PDPK1 overexpression reversed the effects of CPT1A silencing on the viability and glycolysis of PCa cells

**Figure 7.**
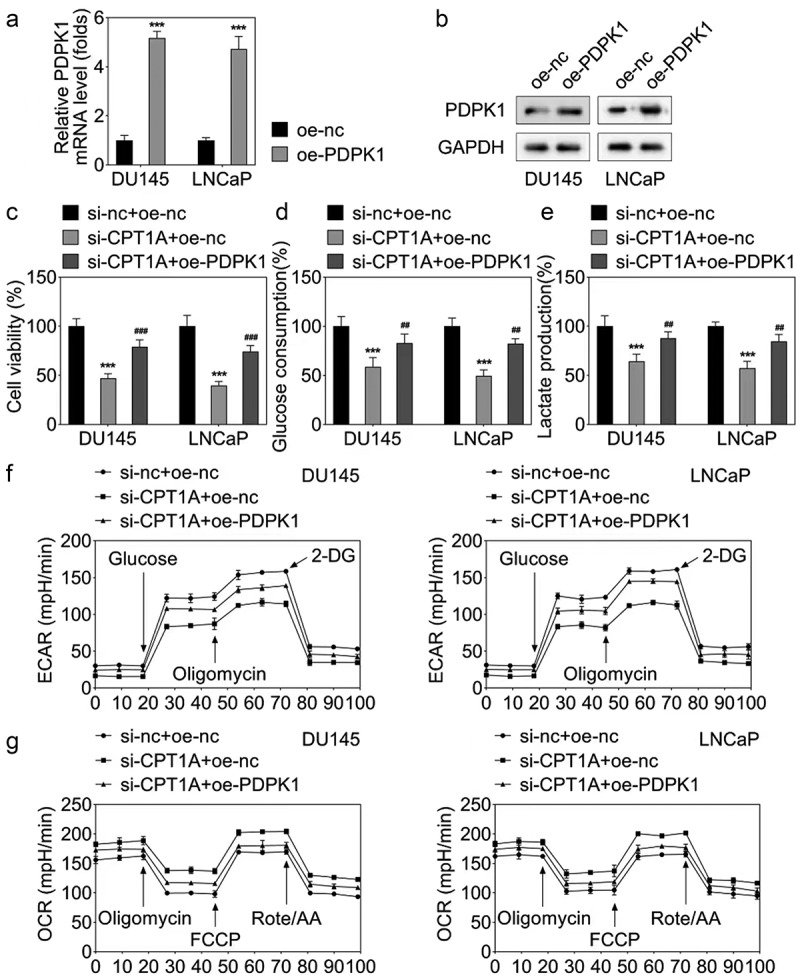
PDPK1 overexpression reversed the effects of CPT1A silencing on the viability and glycolysis of PCa cells. (a) qPCR and (b) western blot were used to detect the expression of PDPK1 after transfection. (c) Cell viability was evaluated by CCK-8 assay. (d,e) glucose consumption and lactate production were measured using commercial kits. (f,g) Seahorse was used to detect the glycolysis of PCa cells after transfection. *p <.001 vs si-nc+oe-nc group, ## *p*  < .01, ### *p*  < .001 vs oe-CPT1A group.

To further confirm the interaction between CPT1A and PDPK1, we performed the rescue experiments. qPCR and western blot results showed that PDPK1 overexpressing vector increased the level of PDPK1 (Figure 7(a,b)). CCK-8 assay showed that PDPK1 overexpression significantly reversed the effect of CPT1A on the viability (Figure 7(c)). The assessment of glucose consumption and lactate production as well as the seahorse evaluation indicated that PDPK1 overexpression reversed the effect of CPT1A on the glycolysis of PCa cells (Figure 7(d–g)).

## Discussion

As previously reported, CPT1A increases reactive oxygen species in the mitochondria and promotes antioxidant defenses in PCa cells.^26^ CPT1A supports castration-resistant PCa by supplying acetyl groups for histone acetylation, promoting growth and antiandrogen resistance.^24^ Here, we confirmed the up-regulation of CPT1A in PCa. Moreover, CPT1A promotes the viability and glycolysis of PCa cells. These findings expanded our understanding of the role of CPT1A in PCa. However, whether regulating the expression of CPT1A has a greater effect on the progression of primary or advanced tumors needs further study. Additionally, CPT1A is a desirable drug target for clinical treatments. Etomoxir, a CPT1A inhibitor, may be a promising drug in the clinical treatment of cancer.^27^ However, whether etomoxir could affect PCa progression remains not understood, which will be studied in our future work.

The correlation analysis combined with experiments determined that PDPK1 was regulated by CPT1A, however, CPT1A did not influence the succinylation of PDPK1. The main mechanism of CPT1A is regulating fatty acid oxidation. For instance, CPT1A-mediated fatty acid oxidation promotes cell proliferation via nucleoside metabolism in nasopharyngeal carcinoma.^28^ Disruption of CPT1A through genetic or pharmacologic ways can cut off the supply of NADPH, thus preventing anchor-independent growth of ESCC cells and lung metastasis.^29^ Recently, CPT1A was identified as a succinyltransferase. Succinylation has gained increasing attention in recent years because succinylation modification is relatively conserved and is involved in almost all biological processes. Succinylation can induce more changes in the physicochemical properties and function of proteins than other PTMS. CPT1A-mediated succinylation of S100A10 increases human gastric cancer invasion.^30^ CPT1A promotes succinylation of MFF at K302, which protects against Parkin-mediated ubiquitin-proteasomal degradation of MFF. And MFF inhibition significantly inhibits the progression of ovarian cancer.^31^ Here, we found that CPT1A bound with SP5 directly and promotes its succinylation. The succinylation of SP5 promotes its binding with the promoter of PDPK1, thus enhancing the PDPK1 transcription.

PDPK1 was identified as an early marker for aggressive PCa which participates in the regulation of PCa progression.^32^ PDPK1 promotes the proliferation of the AR‐negative PCa cells such as DU145 and PC3 via SGK3 activation, independent of the AKT pathway.^33^ Here, we demonstrated that SP5 and HOXA3 both can modulate the transcription of PDPK1 by binding to the promoter region. But CPT1A only modulates the succinylation of SP5 but not HOXA3. SP5 encodes a protein containing a C-terminal Sp1-like zinc finger domain. This zinc finger is highly homologous to that of Sp1 and can bind to the GC box in vitro with the same affinity. The role of SP5 in PCa development remains unknown. We first found that SP5 and HOXA3 mediate the regulation of CPT1A on PDPK1 transcription. The direct effect of SP5 and HOXA3 in PCa progression will be studied in our future work.

PDPK1 plays as a downstream gene of PI3K which is required for the activation of AKT serine/threonine kinase 1. We confirmed here that PDPK1 activates the AKT/mTOR signaling in PCa cells which is consistent with the previous studies.

In conclusion, our findings demonstrate that CPT1A expression is increased in PCa. Silencing of CPT1A exerts tumor-suppressing functions in PCa by suppressing glycolysis. Mechanically, CPT1A promotes the succinylation of SP5 at K391 to transcriptionally activate PDPK1, which activates the AKT/mTOR signal pathway. These findings might provide novel strategies for PCa therapy.

## Data Availability

The datasets used and/or analyzed during the current study are available from the corresponding author on reasonable request.
